# Cold atmospheric plasma jet-generated RONS and their selective effects on normal and carcinoma cells

**DOI:** 10.1038/srep20332

**Published:** 2016-02-03

**Authors:** Sun Ja Kim, T. H. Chung

**Affiliations:** 1 Department of Physics, Dong-A University, Busan 604-714, Republic of Korea

## Abstract

Cold atmospheric helium plasma jets were fabricated and utilized for plasma–cell interactions. The effect of operating parameters and jet design on the generation of specific reactive oxygen and nitrogen species (RONS) within cells and cellular response were investigated. It was found that plasma treatment induced the overproduction of RONS in various cancer cell lines selectively. The plasma under a relatively low applied voltage induced the detachment of cells, a reduction in cell viability, and apoptosis, while the plasma under higher applied voltage led to cellular necrosis in our case. To determine whether plasma-induced reactive oxygen species (ROS) generation occurs through interfering with mitochondria-related cellular response, we examined the plasma effects on ROS generation in both parental A549 cells and A549 ρ^0^ cells. It was observed that cancer cells were more susceptible to plasma-induced RONS (especially nitric oxide (NO) and nitrogen dioxide (NO_2_^−^) radicals) than normal cells, and consequently, plasma induced apoptotic cell responses mainly in cancer cells.

Cold atmospheric plasmas (CAPs) have received great attention due to their remarkable abilities in biological and medical applications^1^,^2^,^3^,^4^,^5^,^6^,^7^. Many groups have successfully demonstrated the potential of CAP as a novel cancer therapy^2^,^8^,^9^,^10^,^11^,^12^,^13^,^14^,^15^,^16^,^17^,^18^. Plasma-induced apoptosis has been observed together with an accumulation of cells in S phase of the cell cycle, which suggests an arrest of tumor proliferation^15^. It has been reported that the ability of plasma to enhance cancer cell death *in vitro* by mitochondria-mediated apoptosis^16^. Further plasma-treatment strategy, such as the advantage of combining plasma with endoplasmic reticulum (ER) stress, has shown that plasma can be selective against cancer cells, not non-malignant cells^18^. Plasma also induces a reduction of tumor volume under *in vivo* studies^15^. Plasmas at atmospheric pressure can involve many active species (charged particles, radicals, UV radiations) that can chemically interact with living materials^1^,^6^. However, the constituent generated by plasma that is linked to this anticancer process and its mechanism of action should be clearly elucidated.

The cellular effects of CAP appear to involve, either directly or indirectly, reactive oxygen and nitrogen species (RONS) created by CAP in air environments^10^. RONS are well recognized for playing a dual role, as they contain both deleterious and beneficial species. The beneficial effects of RONS occur at moderate concentrations and involve physiological roles in cellular responses (e.g., in defense against infectious agents, in the function of a number of cellular signaling pathways, and the induction of mitogenic responses)^19^. In contrast, the overproduction of RONS, including both internal and external generations, results in oxidative stress, which is a deleterious process that can be an important mediator of damage to cell structures, including lipids and membranes, proteins, and DNA. Interestingly, many researchers point out that relatively low doses of plasma appear to induce cell cycle arrest, and higher doses of plasma lead to apoptosis and necrosis in cancer cells^2^,^10^. It seems that these anti-tumor acts of plasma are similar to the consequences of the overproduction of RONS. It is possible that plasma influences the balance between nitrosative and oxidative stress in cells and regulates cellular processes. Generally, active species that exist in and around plasma can cause various effects on cells^20^,^21^. The radical species emitted by a gas–plasma operation are mainly reactive nitrogen species, like nitric oxide (NO) and nitrogen dioxide (NO_2_), as well as reactive oxygen species, like ozone (O_3_), hydroxyl radicals (OH^−^), superoxide (O_2_^−^), and singlet oxygen (_1_O_2_)^22^. Observing the specific causes and impacts of those species will improve the adjustment of treatment doses and allow for the optimization of the plasma process for specific applications. The plasma-generated species that are delivered to the cells depend on the plasma conditions controlled by the design of the source, including the configuration of the electrode. An investigation into the effects of utilized sources on plasma–cell interactions and improvement of the source to maximize the effect would be important for medical applications of atmospheric pressure plasmas. Moreover, the issue of selectivity between normal and cancer cells is also of great significance, because targeting the tumor cells and selectively damaging their functions while leaving normal cells less affected could be an attractive anti-tumor strategy. In order to understand the role of CAP in complex biochemical processes, the plasma-dependent production of reactive species within various target cells and different cellular responses by plasma treatment should be examined carefully.

Although our previous study indicated that the plasma-induced intracellular RONS were observed to increase in cancer cells^23^, it remains unclear whether it can induce apoptotic death effectively and which of these species have a crucial role in the selectivity between normal and cancer cells. We found that the features of cancer cells, maintaining with high basal reactive oxygen species (ROS) levels and low antioxidant capacities^24^, can lead to plasma sensitivity in various cell lines. In order to extend therapeutic application of plasma to cancer treatment, some previous observations^25^,^26^,^27^,^28^ that cancer cells are more susceptible to plasma-induced effects than normal cells require further verification on multiple cell lines and for various plasma sources. Furthermore, the active plasma species among RONS that are mainly responsible for selectivity regarding its action on different types of cells should be clarified.

In healthy eukaryotic cells containing a full complement of mtDNA, cytoplasmic NADH can be efficiently reoxidized by the mitochondrial respiratory system in order to maintain an appropriate cellular redox state. The ablation of the mitochondrial genome, which encodes several key components of the respiratory chain, results in cells that are deficient in mitochondrial respiratory activity^29^. Although the effects of plasma in cellular system have been reported^12^,^13^,^14^,^15^,^16^,^17^,^18^, the mechanism has not fully characterized. The study on the interrelationship between the plasma-effects and the absence of such mitochondrial activity (with rho 0 cells) could be helpful for plasma-medicine study to understand cellular responses under plasma treatment.

A very important issue is the development of appropriate CAP jet devices for cancer therapy. In this study, in order to enhance RONS generation while minimizing the effects of charged particles and UV radiation, CAP jets were fabricated and utilized for plasma treatment on normal and cancer cells. The first aim of this study was to demonstrate that novel CAP jets can induce the overproduction of RONS and apoptosis mainly in cancer cells, providing a therapeutic selectivity between normal and cancer cells. Secondly, we sought to elucidate the mechanism of ROS production by examining whether the increase of plasma-induced ROS also can occur through the mitochondria-independent cellular response.

## Experiment

### Atmospheric pressure helium plasma jet devices

**Figure 1:**
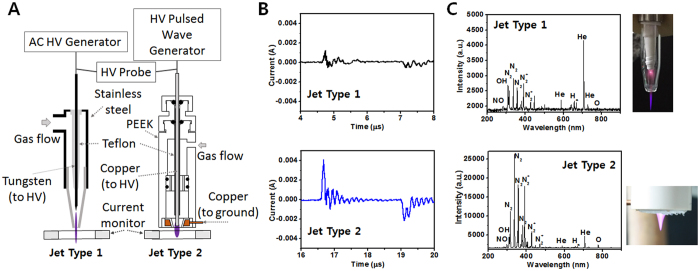
Plasma jet devices and electrical and optical characteristics. (**A**) Schematic of experimental setup and structure of two different jet devices (Jet-Type 1 and 2). (**B**) Waveforms of discharge currents for Jet-Type 1 (upper row of figure) and Jet-Type 2 (bottom row of figure). (**C**) photograph and optical emission spectrum of plasma plume (upper row of figure: Jet-Type 1; bottom row of figure: Jet-Type 2) from 200–900 nm observed in plasma at applied voltage 1.8 kV_pp_, repetition frequency 35 kHz, and duty ratio 8%.

Two different types of jets were utilized for plasma–cell interactions. Figure 1A shows the schematic of the experimental setup and the structure of the jet sources. The plasma jet (Jet-Type 1) consisted of a wire electrode, a Teflon fitting, a glass confinement tube (8-mm inner diameter and 10-mm outer diameter), and a pencil-shaped nozzle (2-mm inner diameter at the exit). A tungsten pin wire (0.3-mm diameter) with a sharpened tip was placed on the tube axis. A pencil-shaped nozzle was attached to the end of the glass tube. The distance between the end of the tip and the glass tube exit was approximately 10 mm. Another kind of plasma jet was fabricated in an attempt to increase the concentration of reactive species and employed to cell treatment. The structure of this improved jet source (Jet-Type 2) is also shown in Fig. 1A. The plasma jet consisted of copper wire electrodes (2-mm diameter), polyetheretherketone (PEEK) plastic housing, and a pencil-shaped grounded-ring electrode (copper, 6-mm inner diameter and 16-mm outer diameter at the exit) covered with dielectric PEEK. The wire was inserted coaxially in the housing and covered with a Teflon tube, leaving a length of 6 mm of the wire exposed to gas (being surrounded by the grounded electrode). The small distance between the powered and grounded electrodes allowed for the local enhancement of the electric field and thus a considerable reduction of the breakdown voltage requirement compared to the plasma jets utilizing the annular ring electrode or dielectric barrier discharge jets.

### Experimental setup with diagnostic system

A sinusoidal voltage of several tens of kilohertz (FT-Lab HPSI200) was applied to the Jet-Type 1, and a pulsed unipolar voltage with a repetition frequency of several tens of kilohertz (FT-Lab PDS 4000) was applied to the Jet-Type 2. The working gas (helium) was delivered at a flow rate of 0.1–2 L/min. The waveforms of the voltage and current were measured using a real-time digital oscilloscope (Lecroy WS44XS-A) via a high-voltage probe (Tektronix P5100) and current monitor (Pearson 4100). To confirm the reactive species generated by the plasma jet in the open air, the emission spectra of the atmospheric pressure plasma jet were monitored using a fiber optic spectrometer (OceanOptics USB-2000 + XR-ES).

### Comparison of characteristics between Jet-Type 1 and 2

Compared to other plasmas sustained in a limited electrode space, CAP jets have the significant advantage of being able to transport reactive species to remote regions for treatment^30^. In order to control plasma properties to produce a higher flux of radicals, it is important to measure the electrical and optical characteristics, such as discharge current, electron density, and emitted spectra. Figure 1B represents the waveforms of the discharge currents in Jet-Type 1 (upper row of figure) and Jet-Type 2 (bottom row of figure). The calculated electron densities were approximately 0.22–0.89 × 10^12^ (Jet-Type 1) and 2.3–6.9 × 10^12 ^cm^−3^ (Jet-Type 2). Jet-Type 2 had a plasma density almost ten times higher than that of Jet-Type 1. Both discharges were generated using a pulsed high-voltage supply, 35 kHz frequency, 8% duty ratio, and 1.7–1.9 kV_pp_ amplitude. The method used to estimate electron density was described in our previous study^31^. Figure 1C shows the photograph of plasma plume and the optical emission spectra recorded from the jets in the wavelength range of 200–900 nm. The optical spectra of plasma exhibited an intensity level from highly reactive radicals (e.g., OH, NO, O, and H_α_) in both jets. However, the richness and enhanced intensity of these species were much stronger in Jet-Type 2 (bottom figure of Fig. 1C) than in Jet-Type 1 (upper figure of Fig. 1C). The electrical and optical characteristics indicated that the newly designed jet source (Jet-Type 2) was more suitable and capable of treating biological materials efficiently than the Jet-Type 1 source due to its increased electron density and enhanced generation of reactive radicals.

## Results and Discussion

### Morphological change and reduction in viability of cells (Using Jet-Type 1)

**Figure 2:**
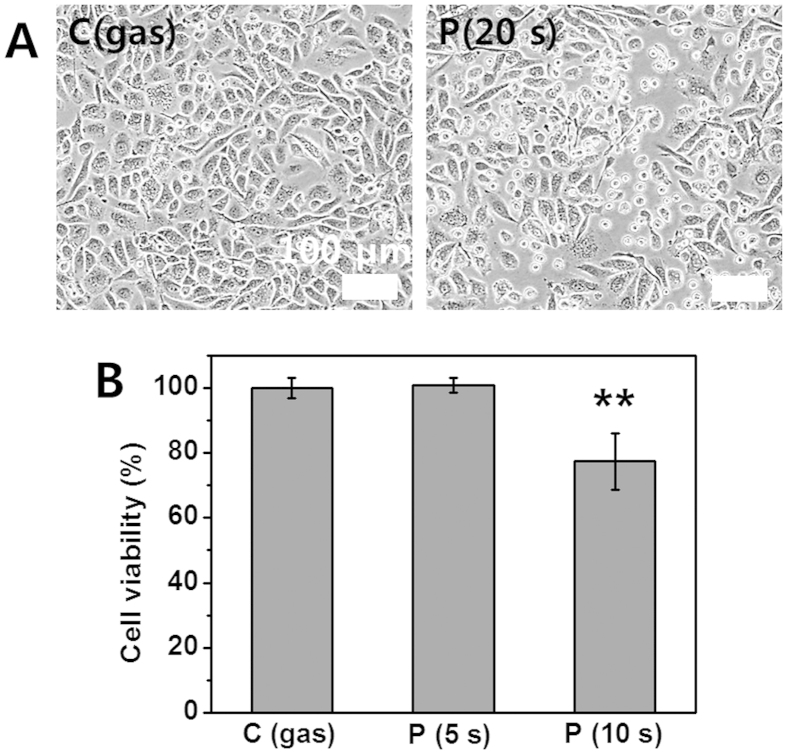
Morphological change and reduction in viability of PC3 cancer cells (using Jet-Type 1). (**A**) Bright field images of cells (left: gas-treated control; right: detachment from surface after plasma treatment (0.8 kV_rms_) for 20 s). (**B**) Measurement of cell viability by MTS assay at 24 h after gas (10 s) and plasma treatment (0.8 kV_rms_) for 5 and 10 s on five points (Each point represents mean ± SD of three replicates; **p < 0.01). Scale bar = 100 μm.

Generally, adherent cells such as prostate cancer cells adhere to culture surfaces via membrane receptors and cell adhesive proteins that reside in the serum or are secreted from the cells in culture^32^. We treated the *in-vitro* prostate cancer cells (PC3; 5 × 10^5 ^cells per well) using the CAP jet (Type 1). It was observed that the cancer cells started to detach from the surface after plasma treatment (Fig. 2A). After cells contact surfaces (passive adhesion stage), cells are always dynamically altering their cell membrane and its morphology to optimize interactions and to stabilize the cell-material surface interface (active adhesion stage)^33^. After plasma-treatment, it was observed that many cells were changed from spread to round cell shapes (similar to passive adhesion stage and trypsin-mediated de-adhesion^34^). It is possible that both adhesive proteins and membrane receptors, such as the extracellular matrix (ECM) and fibronectin around the prostate cancer cells, were disrupted by the plasma treatment. The morphological changes in plasma-treated cells may indicate further involvement of cellular metabolic processes with considerable damages, because the detachment of cells from the ECM can promote apoptosis in a process termed anoikis^25^,^35^. Figure 2B represents the measurement of cell viability by MTS assay on prostate cancer (PC3; 2 × 10^4 ^cells per well) cells. The viability rate of cells was reduced in the plasma-treated cells. It was observed that the rate was lower than that in the gas flow-treated control, and no significant change was observed in 5-s treatment time.

### Intracellular ROS generation (using Jet-Type 1)

**Figure 3:**
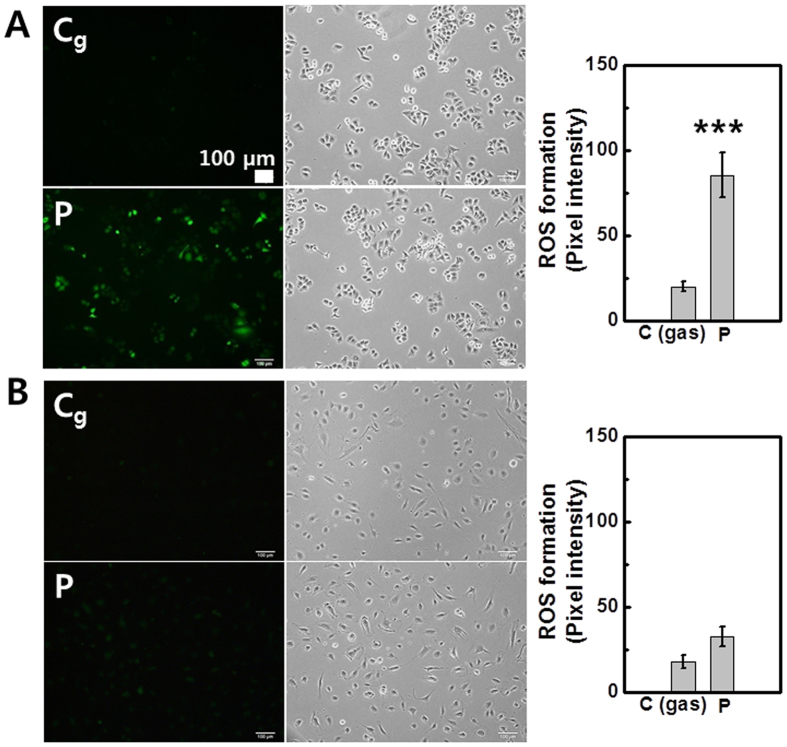
Intracellular ROS generation in cancer (A549) and normal (HCAEC) cells (using Jet-Type 1). Fluorescence images and intensity graphs of intracellular ROS production after plasma treatment for 10 s (0.8 kV_rms_) using DCF-DA assay and bright field images: (**A**) A549 cells (upper row: gas-treated control; bottom row: plasma treatment). (**B**) HCAEC cells (upper row: gas-treated control; bottom row: plasma treatment). Scale bar = 100 μm. (Each point represents mean ± SD of three replicates; ***p < 0.001).

Figure 3 represents the fluorescence images and intensity graphs of intracellular ROS generation induced by plasma treatment in cancer (lung cancer cells; A549; 1 × 10^5 ^cells per well) and normal (human coronary artery endothelial cells; HCAEC; 1 × 10^5 ^cells per well) cell lines using a 2′–7′-dichloro fluorescein diacetate (DCF-DA, Molecular Probes) assay^36^. In the cancer cells after plasma treatment (bottom row of Fig. 3A), the intensity level of the fluorescence was higher than that of gas-treated cells (upper row of Fig. 3A). On the other hand, no significant changes were observed in normal cells (Fig. 3B). The observations indicate that normal and cancer cells have different sensitivities to external plasma exposure. The results also show a plasma-dependent elevation of intracellular ROS production in cancer cells while leaving normal cells less affected. This result indicates that the A549 cancer cells are more responsive to plasma-mediated ROS production than HCAEC normal cells.

### Comparison between parental and ρ^0^ cells in plasma-induced ROS production (using Jet-Type 1)

**Figure 4:**
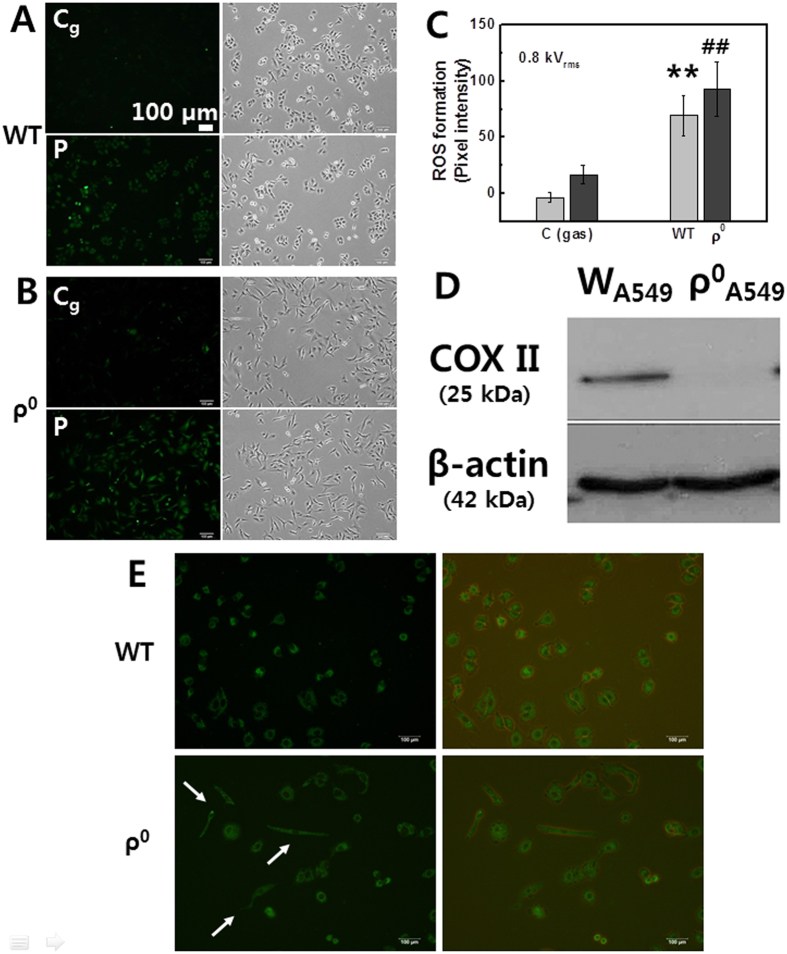
Comparison between parental A549 and A549-ρ^0^ cells in plasma-induced ROS production (using Jet-Type 1). Fluorescence images of intracellular ROS production after plasma treatment for 10 s (0.8 kV_rms_) by using DCF-DA assay and bright field images: (**A**) A549 cells (upper row: gas-treated control; bottom row: plasma treatment) and (**B**) A549-ρ^0^ cells (upper row: gas-treated control; bottom row: plasma treatment). Scale bar = 100 μm. (**C**) Quantification by measuring fluorescence pixel intensity with MetaMorph software (Each point represents mean ± SD of three replicates; **p < 0.01). (**D**) Immunoblot of cytochrome c oxidase II (COX II) showing that this protein, which is coded by mtDNA, is present in A549 cells but not in A549-ρ^0^ cells. Blot was probed with anti-actin to ensure equal protein loading. (**E**) Fluorescence (left) and merged images with bright field image (right) of the mitochondria-staining in A549 cells (upper row) and in A549-ρ^0^ cells (bottom row, morphological change: white arrows).

Mitochondria play a main role in deciding cell fate in intrinsic pathways and are the major source of intracellular ROS under physiological conditions^37^. The anticancer activity of plasma has been reported to involve mitochondrial dysfunction^28^. Cell lines lacking mitochondrial DNA (mtDNA), termed ρ^0^ cells whose mitochondrial function was disabled through the depletion of mtDNA, have been used widely to investigate the relationships between mtDNA mutation, mitochondrial function, and a variety of cellular processes^38^,^39^,^40^,^41^. Long-term treatment of a lung cancer cell line (A549) with ethidium bromide generated mtDNA-deficient ρ^0^ mutants with morphological changes. In order to observe mitochondria-related morphological change, the cell-permeant MitoTracker Green FM probe (Molecular Probe)^42^, which is containing a mildly thiol-reactive chloromethyl moiety for labeling mitochondria, was used. A549 cell normally has a polygonal shape and sheet-like pattern in normal monolayer culture, which is compatible with its epithelial origin^43^. However, the rho 0 cells were changed in cell morphology from a polygonal to an elongated spindle-like shape (Fig. 4E, white arrows). To verify the loss of mtDNA-encoded protein, the expression of mtDNA-encoded cytochrome c oxidase II (COX II) was assessed by Western blotting^40^,^41^. Expression was not observed in ρ^0^ cell lysates as compared to the parental cells (Fig. 4D). To determine whether plasma-induced ROS generation occurs through interfering with mitochondria-related cellular response, we examined the effects of plasma on ROS levels in both parental A549 cells and A549-ρ^0^ cells. Figure 4A,B represent the fluorescence images and intensity graphs of intracellular ROS production and bright field images by using DCF-DA assay in A549 (Fig. 4A) and A549-ρ^0^ (Fig. 4B) cells (1 × 10^5 ^cells per well). The values quantified by measuring pixel intensity with MetaMorph software are shown in Fig. 4C. It was observed that plasma also induced ROS production in A549-ρ^0^ cells, similar to the parental A549 cells. These results suggest that the plasma-mediated ROS may not only result from mitochondria. Mitochondria can also be damaged due to impaired cellular membranes caused by the accumulation of lipid peroxidation products. Further examinations are needed in order to understand the precise mechanisms of both mitochondria-dependent and mitochondria-independent pathways of plasma-mediated effects.

### Induction of necrotic and apoptotic cell death (using Jet-Type 1)

**Figure 5:**
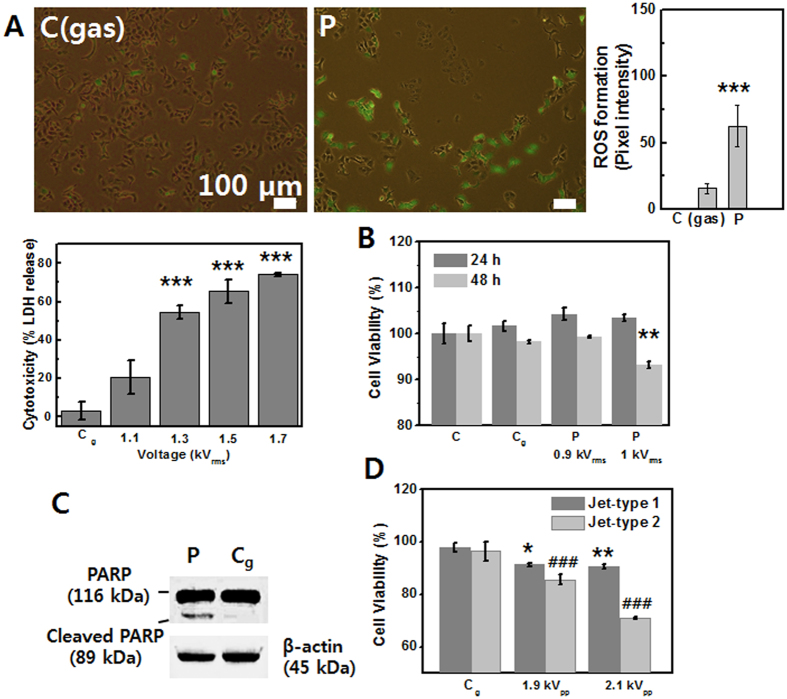
Induction of necrotic and apoptotic cell death in A549 cancer cells (using Jet-Type 1). (**A**) Merged image of fluorescence using DCF-DA with bright field images and intensity graphs after plasma treatment (1.2 kV_rms_) for 10 s (upper row, left: gas-treated control, right: plasma-treatment) and cytotoxicity (% LDH) rate as a function of applied voltage (bottom row). Scale bar = 100 μm. (**B**) Measurement of cell viability by MTS assay at 24 and 48 h after gas and plasma treatment (0.9 and 1 kV_rms_) for 10 s on five points. (**C**) Immunoblot of PARP at 24 h after gas and plasma treatment (1 kV_rms_) for 10 s on nine points per 35-mm dish. Blot was probed with anti-actin to ensure equal protein loading. (**D**) The rate of cell viability by using Jet-Type 1 and 2 under the same parameter with a pulsed high-voltage supply as a function of applied voltage. (Each point represents mean ± SD of three replicates; *p < 0.05, **p < 0.01, ***p < 0.001).

Cellular damage was observed to depend on the plasma dose. The plasma under a relatively low applied voltage induced the detachment of cells, a reduction in cell viability, and apoptosis, while the plasma under higher applied voltage led to cellular necrosis in our case. In order to measure plasma-induced cytotoxicity in A549 cancer cell (2 × 10^4 ^cells per well) as a function of applied voltage, we performed LDH cytotoxicity assay (Takara Shuzo Co)^44^. Cytotoxicity (% LDH release) was dramatically increased with a rise in applied voltage, which indicates a fatal damage of the cell membranes by plasma (Fig. 5A). As shown in the merged fluorescence images of Fig. 5A, plasma-treated cancer (A549; 1 × 10^5 ^cells per well) cells ruptured and left behind considerable cellular debris after detachment. We found that intracellular ROS was produced in surrounding cells (fluorescence intensity graph of Fig. 5A). However, it is desirable that surrounding normal cells are less affected in cancer therapy and that plasma treatment can lead to apoptosis. We focused on inducing apoptosis with rich reactive radical species and controlled plasma parameters in order to avoid necrotic damage. Figure 5B represents the measurement of cell viability in A549 cancer cells (2 × 10^4 ^cells per well). The rate of cell viability was reduced at 48 h after plasma exposure. In order to examine the possibility of apoptosis induction in plasma-treated lung cancer (A549) cells, Western blot analysis was performed. An early biochemical event that accompanies apoptosis in many cell types is the proteolytic cleavage of poly (ADP-ribose) polymerase (PARP) from a 116-kDa polypeptide. PARP has been implicated in the induction of both p53 expression and apoptosis, with the specific proteolysis of the enzyme thought to be a key apoptotic event^45^. As expected, the cleaved fragments of PARP were detected in A549 cells 24 h after plasma treatment (Fig. 5C). In order to compare the efficiency of plasma treatment, we performed MTS assay to show cell viability by using Jet-Type 1 and 2 under the same parameter with a pulsed high-voltage supply as a function of applied voltage (Fig. 5D). The viability rate of cancer (A549; 2  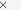 10^4 ^cells per well) cells was reduced in the plasma-treated cells. It was observed that the rate under Jet-Type 2 was much lower than that under Jet-Type 1, which indicates that the impact is more significant in cellular response by using Jet-Type 2.

### Intracellular RONS generation by plasma with varying operating parameters (using Jet-Type 2)

**Figure 6:**
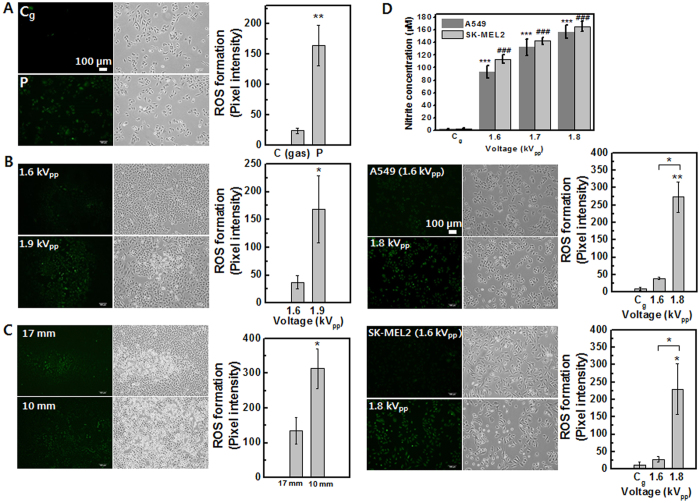
Intracellular RONS generation by plasma under various operating parameters in lung cancer (A549) and melanoma (SK-MEL2) cells (using Jet-Type 2). Fluorescence images and intensity graphs of intracellular ROS production and bright field images: (**A**) DCF-DA assay after plasma treatment (1.7 kV_pp_) (upper row: gas-treated control; bottom row: plasma treatment). (**B**) APF assay as a function of applied voltage (upper row: 1.6 kV_pp_; bottom row: 1.9 kV_pp_). (**C**) DAF-2DA assay after plasma treatment (1.7 kV_pp_) as a function of decreasing distance from nozzle to cells (upper row: 17 mm; bottom row: 10 mm). Scale bar = 100 μm. (**D**) Measurement of nitrite concentration as a function of applied voltage (for 3 min with 3-mm-thick layer of HBSS; 1.6, 1.7, and 1.8 kV_pp_) in A549 and SK-MEL2 cells by Griess assay (upper row) and fluorescence images and intensity graphs of intracellular NO production as a function of applied voltage (1.6 and 1.8 kV_pp_) by DAF-2DA assay (middle row: A549; bottom row: SK-MEL2). (Each point represents mean ± SD of three replicates; *p < 0.05, **p < 0.01, ***p < 0.001).

To fully investigate the effects of RONS on cellular response, a newly fabricated jet (Jet-Type 2) was utilized for plasma–cell interactions. Figure 6 shows the fluorescence images of intracellular RONS production and bright field images including intensity graphs in cancer (A549; 1–5 × 10^5^ cells per well) cells. Figure 6A shows that plasma-treated cell populations containing high levels of DCF fluorescence were increased. In order to specify reactive species more precisely, we used 3′-(p-aminophenyl) fluorescein (APF; Sigma Aldrich)^46^, and likewise, cells were loaded with the fluorescent cell-permeable NO-specific probe 4, 5-diamino-fluorescein diacetate (DAF-2DA, Enzo Life Sciences)^47^. The intracellular production of OH was observed to increase with increasing applied voltage (1.6 kV_pp_ → 1.9 kV_pp_, shown in Fig. 6B). Figure 6C shows the significant enhancement in NO production with decreasing distance from nozzle to cells (17 mm → 10 mm). We used fluorescence probes of APF (Sigma-Aldrich), DAF-2DA (Enzo Life Sciences) and DCFDA (Molecular Probes) for various reactive RONS. DCF-DA is probably the most commonly used reagent for detecting intracellular ROS species, despite its non-specificity and auto-oxidation in the presence of light. The non-fluorescent DCFDA becomes fluorescent in the presence of a wide variety of ROS including, but not limited to, peroxyl (ROO•) and hydroxyl (OH) radicals and the peroxynitrite anion (ONOO^−^). In contrast, APF shows much more limited reactivity and higher resistance to light-induced oxidation^46^. DAF-2DA is also the cell-permeable specific fluorescent NO probe, used to visualize NO synthesis^47^. Due to those features, their intensity was relatively weaker and more confined than DCFDA fluorescent. However, it can also make results specific in terms of plasma-induced RONS in cellular response. In the plasma treatment of living cells, plasma species are delivered to the air-liquid interface and then undergo transportation and sometimes secondary generation of reactive radicals within the liquid medium before reaching cells^48^. In order to measure NO production in liquid-cell phase after plasma treatment, the Griess reaction can be used to analyze nitrite and can provide a good measure of the nitric oxide production in aqueous solutions^49^,^50^. The Griess assay kit (Molecular Probes) was used to analyze nitrite concentration after plasma treatment in lung (A549; 3 × 10^4 ^cells per well) and skin (SK-MEL2; 3  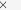
10^4 ^cells per well) cancer cells. Figure 6D shows the results of the NO quantitation assay performed on cells containing serum-free Hanks’ balanced salt solution (HBSS) (layer 3 mm). No significant difference was observed in gas-only exposure control. On the other hand, both cell types exhibited increases in nitrite concentration with increasing applied voltage after plasma exposure. These results have a correlation with the intracellular production of NO (performed DAF-2DA assay) by plasma treatment (the fluorescent images and intensity graphs of Fig. 6D).

### Induction of cellular apoptotic-like change (using Jet-Type 2)

**Figure 7:**
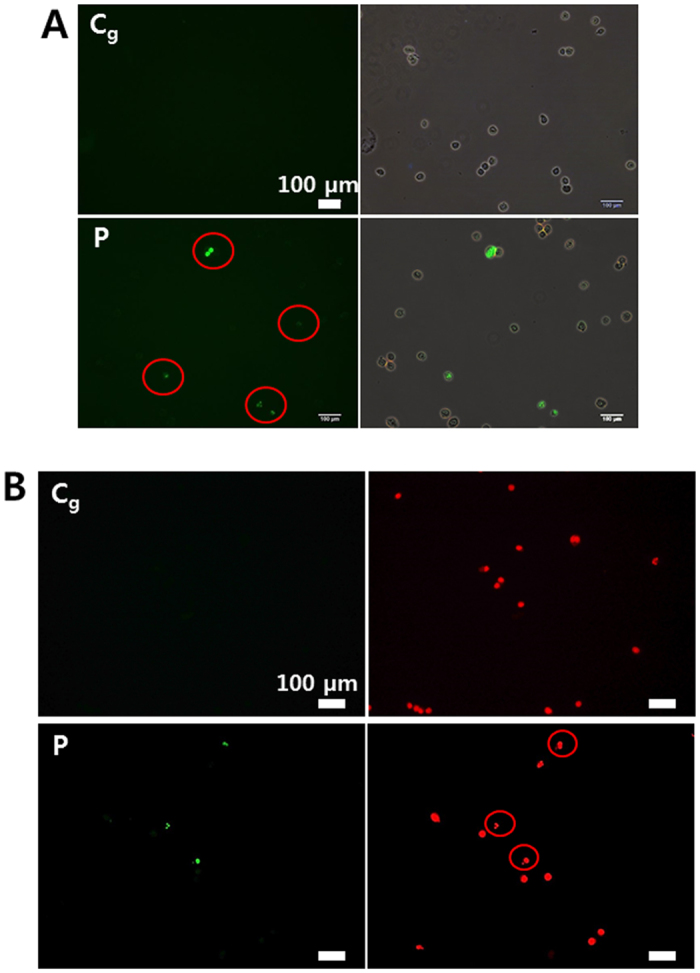
Induction of cellular apoptotic-like change (using Jet-Type 2). (**A**) TUNEL-positive (green) apoptotic cells (red circles) and merged images (upper row: gas-treated control; bottom row: plasma treatment) by TUNEL assay at 48 h after plasma treatment (1.9 kV_pp_) for 5 min on a dish (with 3-mm-thick layer of serum-free media). Cells were harvested by trypsinization 48 h after exposure to plasma and then fixed with 1% paraformaldehyde for 20 min in PBS. (**B**) The images with TUNEL-positive (green) apoptotic cells and cell nuclei (red) by PI staining (upper row: gas-treated control, bottom row: plasma-treatment). Cells stained weakly and irregularly by PI (red circles), indicating that these nuclei were fragmented after plasma exposure. Scale bar = 100 μm.

We examined plasma-induced cellular apoptotic-like change using the TUNEL technique in cancer (A549) cells. Cell nuclei by propidium iodide (PI) staining were also observed with microscopy. As shown in Fig. 7A, the ratio of TUNEL-positive cells (bottom row of figure) increased after plasma treatment compared to that in gas-treated control cells (upper row of figure). Apoptosis can be analyzed by staining with PI, which is known to stain intact nuclei strongly but apoptotic ones irregularly and weakly^51^. It was observed that TUNEL-positive cells were stained weakly and irregularly by PI (Fig. 7B), indicating that these nuclei were fragmented after plasma exposure.

### Level of intracellular OH and NO production in cancer and normal cells (using Jet-Type 2)

**Figure 8:**
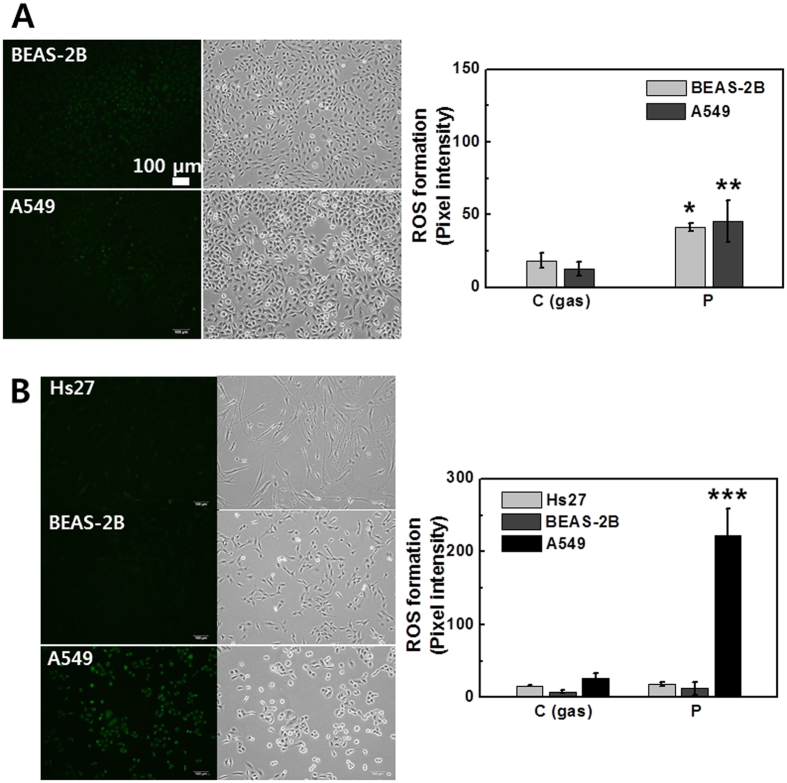
Level of intracellular OH and NO production in cancer and normal cells (using Jet-Type 2). Fluorescence images and intensity graphs of intracellular ROS production after plasma treatment for 10 s (1.7 kV_pp_) and bright field images: (**A**) APF assay (upper row: normal BEAS-2B; bottom row: cancer A549). (**B**) DAF-2DA (upper row: normal HS27; middle row: normal BEAS-2B; bottom row: cancer A549). Scale bar = 100 μm. (Each point represents mean ± SD of three replicates; ***p < 0.001).

Cancer cells are usually under oxidative stress and have a relatively high basal level of RONS. A small induction of RONS in tumor cells may push the level of ROS over the threshold to induce cell death, whereas normal cells can better tolerate the oxidative insults because of their lower basal level of RONS and stronger antioxidant capacities^24^. Inducing RONS generation by plasma has become a novel approach to treating cancer cells^1^,^10^. However, selectively inducing cancer cells to die remains a considerable issue and challenge for cancer treatment. To investigate the selective impact of plasma, we analyzed the effects of RONS production on both tumor cells and normal cells. The observations indicated that the intracellular production of OH and ^–^OCl was similar in both normal (Beas-2B; 3  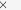 10^5 ^cells per well) and cancer (A549; 3 × 10^5 ^cells per well) cells (Fig. 8A). However, the production of NO was different between normal (Hs27 and Beas-2B; 1 ×10^5 ^cells per well) and cancer (A549; 1 × 10^5 ^cells per well) cells (Fig. 8B), indicating that the A549 cancer cells are more responsive to plasma-mediated NO production than Hs27 fibroblast and Beas-2B epithelial normal cells. NO has several cytotoxic effects, including reactions with proteins and nucleic acids. NO-mediated apoptosis is often observed in pathological cases^52^. Understanding the role of CAP in generating NO_x_-containing species that may be able to interact with complex biochemical processes will lead to extended applications in plasma cancer therapy.

### Measurement of nitrite concentration and caspase 3/7 activation in normal and cancer cells (using Jet-Type 2)

**Figure 9:**
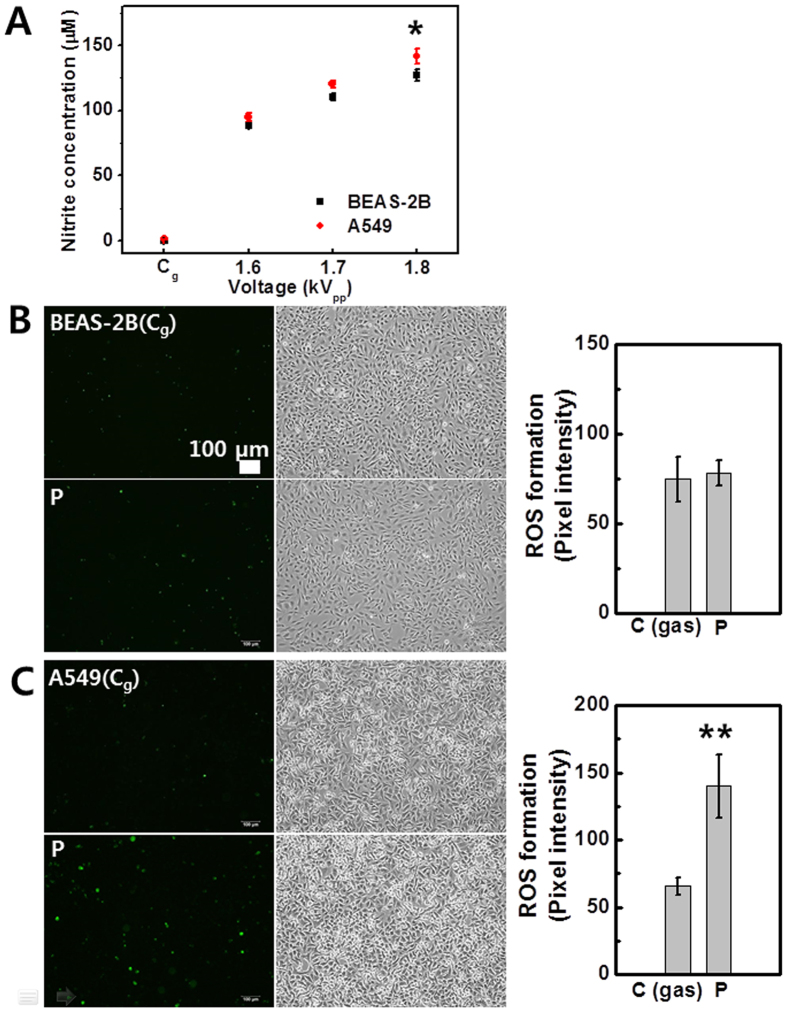
Measurement of nitrite concentration and caspase 3/7 activation in cancer and normal cells (using Jet-Type 2). (**A**) Measurement of nitrite concentration as a function of applied voltage (1.6, 1.7, and 1.8 kV_pp_) in cancer A549 and normal BEAS-2B cells by Griess assay at 12 h after treatment for 3 min with 3-mm-thick layer of HBSS (each point represents mean ± SD of three replicates). Fluorescence images and intensity graphs of caspase 3/7 activation and bright field images in (**B**) BEAS-2B (upper row: gas-treated control; bottom row: plasma treatment) and (**C**) A549 cells (upper row: gas-treated control; bottom row: plasma treatment). Cells were loaded with CellEvent Caspase 3/7 Green Detection Reagent at 16 h after plasma treatment (1.8 kV_pp_) for 10 s. Positive apoptotic cells containing active caspases 3 and 7 appear in green. Scale bar = 100 μm. (Each point represents mean ± SD of three replicates; *p < 0.05, **p < 0.01).

Figure 9A shows the measurement of nitrite concentration with increasing applied voltage in normal (Beas-2B; 3 × 10^4 ^cells per well) and cancer (A549; 3 × 10^4 ^cells per well) cells at 12 h after plasma treatment by Griess assay. The detected nitrite concentration was higher in cancer cells compared to that in normal cells. NO_2_^−^ and NO_3_^−^ are the metabolic products of NO and are convenient markers of NO formation^53^. This result shows that although both cells were under NO-mediated oxidant condition by plasma, cancer cells were more affected (Fig. 9A). Detection of plasma-mediated apoptosis in both normal (Beas-2B; 5 × 10^5 ^cells per well) and cancer (A549; 5 × 10^5 ^cells per well) cells was also performed using the CellEvent Caspase 3/7 Green Detection Reagent, a substrate that becomes fluorescent upon cleavage by active caspase 3 and 7^54^,^55^. Cells were loaded 16 h after treatment. Interestingly, no difference was observed between gas-control and plasma-treated cells in normal cells (Fig. 9B), whereas the activation was observed only in cancer cells after treatment (Fig. 9C). The results support the hypothesis that NO production by plasma may correlate with apoptosis in cancer cells and plasma-induced effects demonstrate the selectivity against tumor cells.

In conclusion, it has been demonstrated that a newly developed CAP jet induces the overproduction of RONS and apoptotic-like changes in cancer cells. The rate of cell viability under Jet-Type 2 was much lower than that under Jet-Type 1, which indicates that the impact is more significant in cellular response by using Jet-Type 2. This is attributable to the increased plasma density and enhanced production of extracellular reactive species. We revealed that different cancer cells exhibited diverse plasma sensitivity. Fluorescence probes of APF (mainly OH detection) and DAF-2DA (NO detection) had much more specific and confined activity than DCFDA (most ROS detection) in plasma-mediated RONS production. After plasma-treatment, various cancer cells were changed from spread to round cell shapes and overproduced the intracellular RONS. Moreover, cytotoxicity was dramatically increased with a rise in applied voltage, which suggests a fatal damage of the cell membranes by plasma. Recently, an important issue of CAP interaction in the anti-cancer therapy is selectivity^56^. The use of RONS near the ‘threshold’ between levels of RONS in normal and cancer cells can allow the selectivity toward tumor cells^56^. The cancer cells were more susceptible to plasma-induced RONS, especially NO and NO_2_^−^ radicals, than normal cells, and consequently, plasmas induced apoptotic-like cellular responses mainly in cancer cells, providing a therapeutic selectivity between tumor and normal cells. A549-ρ^0^ cells depleted of mtDNA were generated and utilized to observe the interrelationship between the plasma-effects and the absence of such mitochondrial activity. This idea could be helpful for plasma-medicine study to understand mitochondria-related cellular responses under plasma treatment. Mitochondria have been identified as a major source of cellular ROS generation^12^. However, it was observed that the increase of plasma-induced ROS can occur through the mitochondria-independent cellular response. This result could suggest plasma-induced non-mitochondrial ROS sources in cancer treatment. We expect that our studies can help elucidate the potential applications of anti-cancer CAP strategies. Although the apoptosis induced by plasma-generated ROS has been considered a promising anti-cancer therapy, the issue of treating tumor cells selectively on multiple cell lines and their underlying mechanisms should be further investigated.

## Methods

### Cell culture

The interaction of the plasma jet with living cells was examined in human prostate cancer cells (PC3; between passage numbers 10 and 15), lung adenocarcinoma cells (A549; between passage numbers 3 and 15), melanoma cells (SK-MEL2; between passage numbers 4 and 20), HCAEC (primary, between passage numbers 1 and 3) cells, foreskin fibroblast (Hs27; between passage numbers 3 and 8) cells, and bronchial epithelial cells (BEAS-2B; between passage numbers 3 and 15). The cells were maintained in F-12K Medium (Kaighn’s Modification of Ham’s F-12 Medium; PC3), DMEM (Dulbecco’s Modification of Eagle’s Medium; A549, Hs27), EGM (Endothelial Cell Growth Medium with VEGF; HCAEC), BEGM (Bronchial Epithelial Cell Basal Medium and the growth supplements; BEAS-2B), EMEM (Eagle's Minimum Essential Medium; SK-MEL2) and supplemented with 10% fetal bovine serum (FBS) and 100 U/ml of penicillin. Cells were incubated at 37 °C with humidified air and 5% CO_2_. For sample preparation, cells were trypsinized and transferred to 60-mm Petri dishes (or 35-mm dishes).

### Generation of Rho 0 cells (A549-ρ^0^)

A549-ρ^0^ cells depleted of mtDNA were generated by incubating wild-type cells (A549) for 4–6 weeks with 50 ng/ml ethidium bromide. The medium was supplemented with 4.5 mg/ml glucose, 50 μg/ml uridine, and 100 μg/ml pyruvate to compensate for the respiratory metabolism deficit^38^. After selection, the ρ^0^ cells were cultured in the above-specified medium without ethidium bromide.

### Plasma–cell interaction

The applied voltage, excitation frequency, and gas flow rate were 0.8–1.2 kV_rms_, 35 kHz, and 0.1 L/min, respectively (Jet-Type 1). A typical operating condition of the pulsed dc plasma jet has an applied voltage of 1.7 kV_pp_, repetition frequency of 35 kHz, gas flow rate of 2 L/min, and duty ratio of 8%, unless otherwise stated (Jet-Type 2). Prior to plasma treatment, media from each chamber was almost completely removed, and a small amount of serum-free HBSS (a few hundred microliters) was left to keep cells wet during treatment. Cells were exposed to plasma (and/or gas flow only) for 10 s on nine designated points per dish (detection of intracellular RONS, Western blot with anti-PARP, and activation of caspase-3/7), for 10 s on five points per 24-well plate (serum-free media, MTS and LDH assay), for 5 min on a dish (with 3-mm-thick layer of serum-free media, TUNEL assay), and for 3 min on a dish (with 3-mm-thick layer of HBSS, Griess assay). The distance from the nozzle to the cell surface was between 10 and 17 mm.

### Measurement of intracellular RONS

The intracellular generation of ROS after plasma treatment was detected by fluorescence microscopy using DCF-DA. The intracellular ROS assay is a cell-based assay for measuring reactive oxygen species activity within a cell. The assay employs a cell-permeable fluorogenic probe DCF-DA (Molecular Probes), which diffuses into cells and is deacetylated by cellular esterases to non-fluorescent DCFH. In the presence of ROS, DCFH is rapidly oxidized to become highly fluorescent DCF^36^. Thus, the generation of ROS can be detected by monitoring the increase in fluorescence. APF has much more limited reactivity and higher resistance to light-induced oxidation; namely, the fluorescein derivative is non-fluorescent until it reacts with the hydroxyl radical or peroxynitrite anion^46^. In order to examine more specific reactive species, we used APF (10 μM), and likewise, cells were loaded with the fluorescent cell-permeable NO-specific probe (DAF-2DA; 10 μM). DAF-2DA and APF were also performed using the same procedure that was used for the DCF-DA assay. Fluorescence was measured with excitation and emission wavelengths set at 488 and 520 nm, respectively. Cells in dishes (Corning diameter 60 mm) were pretreated with 10-μM assay reagent for 5 min at 37 °C in the dark. Then, cells were exposed to plasma (and/or gas flow only) for 10 s on nine points per dish and incubated for 5 min. Intracellular ROS production was observed on the marked points. After plasma treatment, cells were washed with phosphate-buffered saline (PBS). Fluorescence-activated cells were detected using a fluorescence microscope (Nikon) and quantified by measuring pixel intensity with MetaMorph software (Molecular Devices, Sunnyvale, CA).

### MTS assay

Cell viability was assessed by MTS assay with the use of a kit (Promega) according to the manufacturer’s instructions. The MTS tetrazolium compound is bio-reduced by cells into a colored formazan product that is soluble in tissue culture medium. This conversion is presumably accomplished by the NADPH (nicotinamide adenine dinucleotide phosphate; reduced form) or NADH (nicotinamide adenine dinucleotide; reduced form) produced by the dehydrogenase enzymes in metabolically active cells^57^. Assays are performed by adding a small amount of the solution reagent directly to culture wells, incubating for 2–4 h, and then recording the absorbance at 490 nm with the Victor3 spectrophotometer (Perkin-Elmer, CT).

### LDH cytotoxicity assay

The LDH Cytoxicity assay is a colorimetric measure of cell cytotoxicity/cytolysis based on the measurement of LDH activity in the cell culture supernatant. LDH is a stable cytoplasmic enzyme and released into culture supernatant upon damage of the cell membranes^44^. LDH activity was assessed by LDH cytotoxicity detection kit (Takara Shuzo Co), according to the manufacturer's instructions. The supernatant was carefully removed and transferred into an optically clear 96-well plate at 6 h after plasma treatment. The absorbance at 490 nm was measured using the Victor3 spectrophotometer (Perkin-Elmer, CT) at 30 min after adding the reaction solution provided by the manufacturer. The relative LDH release was calculated by the ratio of LDH release over control samples. Controls were treated with 1% Triton X-100 and set as 100% LDH release and 0% for cells with culture medium only.

### Western blot analysis

The expression of proteins was evaluated using Western blot analysis. Whole-cell lysates were prepared using 1× lysis buffer (Cell Signaling). The protein concentration was measured by using a bicinchoninic acid (BCA) protein assay kit (Thermo Scientific). Equivalent amounts of protein (40 μg) from each lysate were resolved by 10% SDS-PAGE (sodium dodecyl sulfate polyacrylamide gel electrophoresis) gels, transferred to polyvinylidene difluoride (PVDF) membranes (Whatman) by electroblotting, and then probed with COXII (Molecular Probes), PARP, and β-Actin (as a loading control) antibodies (Cell Signaling Technology). Membranes were incubated with secondary horseradish peroxidase (HRP)-conjugated antibody (Santa Cruz Biotechnology). Blots were developed using enhanced chemoluminescence (ECL; Pierce).

### Griess assay

The Griess reaction is a technically simple method for the analysis of nitrites in aqueous solutions. It is the use of the Griess diazotization reaction to spectrophotometrically detect nitrite formed by the spontaneous oxidation of NO under physiological conditions. Sulfanilic acid is quantitatively converted to a diazonium salt by reaction with nitrite in acid solution. The diazonium salt is then coupled to N-(1-naphthyl)ethylenediamine, forming an azo dye that can be spectrophotometrically quantitated based on its absorbance at 548 nm^58^. The dish plate (diameter 20 mm) including cells was irradiated with plasma, and samples of the liquids were taken from dish after plasma treatment using a 96-well plate to perform the Griess assay. The absorbance at 548 nm was measured using a Victor3 spectrophotometer (Perkin-Elmer, CT) following incubation (usually 30–45 min) after plasma treatment (3 min). In order to quantify the nitrite concentrations, a calibration curve was prepared using the standard sodium nitrite solutions (Molecular Probes).

### TUNEL assay

TUNEL is an assay used for the localization of apoptotic DNA fragmentation^59^. The method relies on the template-independent identification of blunt ends of double-stranded DNA breaks by TdT. The enzyme catalyzes the addition of labeled dUTPs to a 3′-hydroxyl termini of DNA ends, which can be visualized using immunohistochemical techniques^60^. The extent of apoptosis was assessed with the APO-BrdU (deoxythymidine analog 5-bromo-2′-deoxyuridine 5′-triphosphate) TUNEL assay kit (Molecular Probes). Fragmentation of DNA in apoptotic cells is measured by BrdU incorporation, which is visualized by conjugation to an Alexa Fluor 488 dye-labeled anti-BrdU antibody. Cells were harvested by trypsinization 48 h after exposure to plasma and then fixed with 1% paraformaldehyde for 20 min in PBS. After fixation, the TUNEL assay was performed by following the manufacturer’s instructions. The emitted green fluorescence of apoptotic cells was observed under a fluorescence microscope (Nikon).

### Detection of caspase 3/7 activation

We used a fluorogenic and sensitive reagent (CellEvent Caspase-3/7 Green reagent, Molecular Probes) for the detection of cells destined for cell death. It is a four-amino-acid peptide (DEVD) conjugated to a nucleic acid-binding dye that is non-fluorescent when not bound to DNA. The CellEvent Caspase-3/7 Green reagent is intrinsically non-fluorescent, as the DEVD peptide inhibits the binding of the dye to DNA^54^,^55^. Upon activation of caspase-3/7 in apoptotic cells, the DEVD peptide is cleaved, and the free dye can bind DNA, generating a bright green fluorescence. It is excited with a maximum at 502 nm and has an emission maximum at 530 nm. The emitted green fluorescence of apoptotic cells was observed under a fluorescence microscope (Nikon).

### Statistical analysis

The results were expressed as means ± standard deviation (SD). Statistical significance of difference between groups was analyzed by one-way analysis of variance (ANOVA) followed by Tukey’s multiple comparison test using statistical software (Prism,version 4; GraphPad Software Inc., San Diego, CA, USA).

## Additional Information

**How to cite this article**: Kim, S. J. and Chung, T. H. Cold atmospheric plasma jet-generated RONS and their selective effects on normal and carcinoma cells. *Sci. Rep*. **6**, 20332; doi: 10.1038/srep20332 (2016).
